# RIG-like Helicase Regulation of Chitinase 3-like 1 Axis and Pulmonary Metastasis

**DOI:** 10.1038/srep26299

**Published:** 2016-05-20

**Authors:** Bing Ma, Erica L. Herzog, Meagan Moore, Chang-Min Lee, Sung Hun Na, Chun Geun Lee, Jack A. Elias

**Affiliations:** 1 Department of Molecular Microbiology and Immunology, Brown University, Providence, R.I. 02912, USA; 2 Yale University School of Medicine, Section of Pulmonary and Critical Care Medicine, Department of Internal Medicine, 300 Cedar Street (TAC S441), New Haven, CT 06520, USA; 3 Division of Medical and Biological Sciences, Warren Alpert Medical School, Brown University, Providence, R.I. 02912, USA.

## Abstract

Chi3l1 is induced by a variety of cancers where it portends a poor prognosis and plays a key role in the generation of metastasis. However, the mechanisms that Chi3l1 uses to mediate these responses and the pathways that control Chi3l1-induced tumor responses are poorly understood. We characterized the mechanisms that Chi3l1 uses to foster tumor progression and the ability of the RIG-like helicase (RLH) innate immune response to control Chi3l1 elaboration and pulmonary metastasis. Here we demonstrate that RLH activation inhibits tumor induction of Chi3l1 and the expression of receptor IL-13Rα2 and pulmonary metastasis while restoring NK cell accumulation and activation, augmenting the expression of IFN-α/β, chemerin and its receptor ChemR23, p-cofilin, LIMK2 and PTEN and inhibiting BRAF and NLRX1 in a MAVS-dependent manner. These studies demonstrate that Chi3l1 is a multifaceted immune stimulator of tumor progression and metastasis whose elaboration and tissue effects are abrogated by RLH innate immune responses.

The prototypic chitinase-like protein (CLP), Chitinase 3-like-1 (Chi3l1) (also called as YKL-40 in humans and BRP-39 in rodents) is a member of the 18 glycosyl hydrolase (GH 18) gene family, which binds to but does not degrade chitin^1^. The retention of GH 18 moieties over species and evolutionary time has led to the belief that these moieties play essential roles in biology^2^,^3^. In support of this speculation, recent studies from our laboratory and others demonstrated that Chi3l1 plays a major role in anti-pathogen, antigen and oxidant-induced inflammatory, repair and remodeling responses by regulating a variety of essential biologic processes including oxidant injury, apoptosis, pyroptosis, inflammasome activation, Th1/Th2 cytokine balance, M2 macrophage differentiation, TGF-β1 elaboration, dendritic cell accumulation and activation, fat accumulation and the activation of MAPK, Akt and Wnt/β-catenin signaling^4^,^5^,^6^,^7^,^8^,^9^,^10^,^11^. Studies from our laboratory and others have demonstrated that Chi3l1 is expressed by a variety of cells including macrophages, and epithelial cells and is stimulated by a number of mediators including IL-13, IL-6, IL-1β, and IFN-γ^7^,^8^. These studies also identified significant correlations between dysregulated Chi3l1 and the development, severity and/or progression of a number of diseases including asthma, pulmonary fibrosis and obesity (as reviewed in references^1^,^12^). Chi3l1 dysregulation is particularly striking in solid tumors with the levels of circulating Chi3l1/YKL-40 being increased in patients with cancers of the lung, prostate, colon, rectum, ovary, kidney, breast, glioblastomas and malignant melanoma where they correlate directly with disease progression and inversely with disease free interval and patient survival^12^,^13^,^14^,^15^,^16^,^17^,^18^,^19^,^20^,^21^. These studies strongly suggest that Chi3l1 plays an important role(s) in the biology that underlies these malignancies. However, the mechanisms that Chi3l1 uses to contribute to tumor progression have not been adequately defined.

Metastatic spread is an ominous prognostic event in cancer biology. This can be readily appreciated in malignant melanoma where there is a good chance of recovery if the primary lesion is detected early and the 5 year survival is less than 10% in patients with distant melanoma metastases (stages III and IV)^22^. Malignant melanoma is one of the most aggressive forms of cancer, accounts for 80% of skin cancer deaths and is increasing faster than any other malignancy^22^,^23^. Patients with malignant melanoma have increased levels of circulating Chi3l1/YKL-40 which have been shown to be a risk factor for disease progression^17^,^20^. Recent studies from our laboratory also demonstrated that the induction of Chi3l1 is a critical event in the generation of a metastasis permissive environment^24^. They also demonstrated that this induction is mediated by a novel pathway in which semaphorin 7a (Sema7a) stimulates Chi3l1 by interacting with its β1 integrin receptor^24^. However, despite the importance of tumor associated Chi3l1, interventions that inhibit Chi3l1 have not been adequately described and the ability of these interventions to control the progression of melanoma and other tumors has not been defined.

We hypothesized that interventions that alter the induction of Chi3l1/YKL-40/BRP-39 decrease the metastatic spread of malignant melanoma and other tumors. We also hypothesized that the RIG-like helicase (RLH) innate immune response is a powerful inhibitor of Chi3l1 and pulmonary metastasis. To test these hypotheses we characterized the ability of the known pulmonary RLH activator Poly(I:C)^25^,^26^,^27^ to regulate Chi3l1 production and melanoma and breast cancer metastasis and the mechanisms that are involved in these regulatory events. These studies demonstrate that RLH activation decreases Chi3l1 expression and pulmonary metastasis while regulating NK cell function and activation, cofilin phosphorylation and LIMK2, PTEN, BRAF and NLRX1 expression via a mitochondrial antiviral signaling protein (MAVS)-dependent pathways.

## Results

### Poly(I:C) inhibits pulmonary metastasis

**Figure 1:**
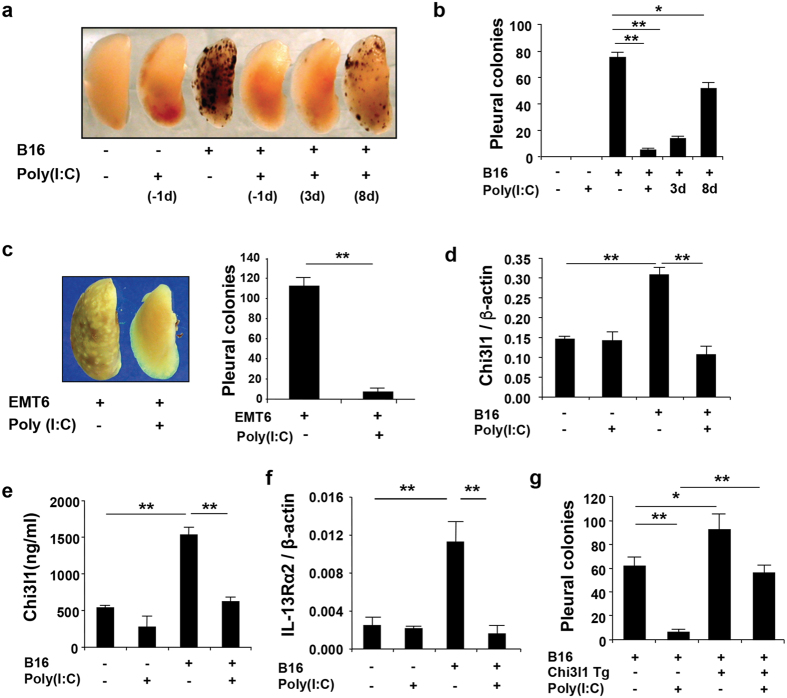
Poly(I:C) inhibits pulmonary metastasis and Chi3l1. (**a**) WT mice were given B16-F10 (B16) melanoma cells or control vehicle, treated with Poly(I:C) or vehicle control and evaluated 2 weeks later. Representative lungs from mice treated with Poly(I:C) one day before (−1d) and 3 (3d) and 8 (8d) days after B16 challenge as indicated. (**b**) The number of pleural melanoma colonies in lungs from mice treated as described in panel **a**. (**c**) Representative lungs and pleural EMT6 cell colony quantification in mice treated with (+) or without (−) Poly(I:C). (**d,e**) The levels of Chi3l1 mRNA and protein in lungs from WT mice and B16 challenged mice treated with Poly(I:C) or vehicle control. (**f** ) The levels of expression of IL-13Rα2 in lungs from unchallenged (−) and B16 challenged (+) WT mice treated with Poly(I:C) or control vehicle. (**g**) The number of pleural melanoma colonies in lungs from WT and Chi3l1/YKL-40 transgenic mice (Tg+) treated with Poly(I:C) or vehicle. Panels **a**,**c** are representative of at least 5 similar evaluations. The plotted values in panels **b**–**g** represent the mean ± SEM of evaluations with a minimum of 4 mice. *P < 0.05. **P < 0.01.

As shown in the previous studies from our laboratory^24^, intravenously administered malignant melanocytes readily form metastatic colonies in the lung (Fig. 1a,b). To determine if treatment with Poly(I:C) altered this response we compared the metastasis in wild type (WT) mice treated with Poly(I:C) or vehicle every other day starting the day before or at intervals after the intravenous administration of B16-F10 (B16) melanoma cells. Poly(I:C) was an effective inhibitor of melanoma metastasis in WT mice when it was administered one day before the malignant cells. Importantly, the effects of Poly(I:C) were not restricted to this pretreatment protocol because Poly(I:C) also inhibited melanoma metastasis when administered 3 or 8 days after the melanoma cells (Fig. 1a,b). This effect was also not melanoma-specific because the metastasis of EMT6 breast cancer cells to the lung was similarly decreased in WT mice that were treated with Poly(I:C) (Fig. 1c). When viewed in combination, these studies demonstrate that pulmonary Poly(I:C) inhibits pulmonary melanoma and other metastasis when administered before or after the malignant cells gain access the vascular space.

### Poly(I:C) inhibits pulmonary metastasis by regulating Chi3l1

Because Chi3l1 plays a critical role in the pathogenesis of pulmonary metastasis^24^, studies were next undertaken to determine if Poly(I:C) altered the ability of melanoma cells to induce Chi3l1. In these experiments we evaluated the expression of Chi3l1 in tumor inoculated WT mice treated with Poly(I:C) or vehicle control. These *in vivo* studies demonstrated that Chi3l1 expression and production are augmented after the administration of B16 melanoma cells and that Poly(I:C) is a potent inhibitor of these metastasis permitting responses (Fig. 1d,e). Melanoma cell challenge also augmented and Poly(I:C) inhibited the expression of the Chi3l1 receptor IL-13Rα2 (Fig. 1f). Further support for the concept that Poly(I:C) inhibits melanoma metastasis, at least in part, by its ability to inhibit the production of Chi3l1 comes from studies comparing the inhibitory effects of Poly(I:C) in mice in which Chi3l1 is regulated normally and mice in which Chi3l1/YKL-40 is expressed solely in the lung (Chi3l1 Tg) using a Poly(I:C)-insensitive promoter (data not shown)^25^,^26^,^27^. In these experiments the transgenic overexpression of Chi3l1/YKL-40 in Chi3l1 null mice significantly opposed the ability of Poly(I:C) to inhibit melanoma metastasis (Fig. 1g). These studies demonstrate that Poly(I:C) inhibits pulmonary metastasis via a Chi3l1/BRP-39-dependent mechanism(s) because its inhibitory effects are associated with the inhibition of Chi3l1 and ameliorated by the overexpression of Chi3l1/YKL-40.

### Poly(I:C) utilizes the RLH pathway to inhibit Chi3l1 and pulmonary metastasis

**Figure 2:**
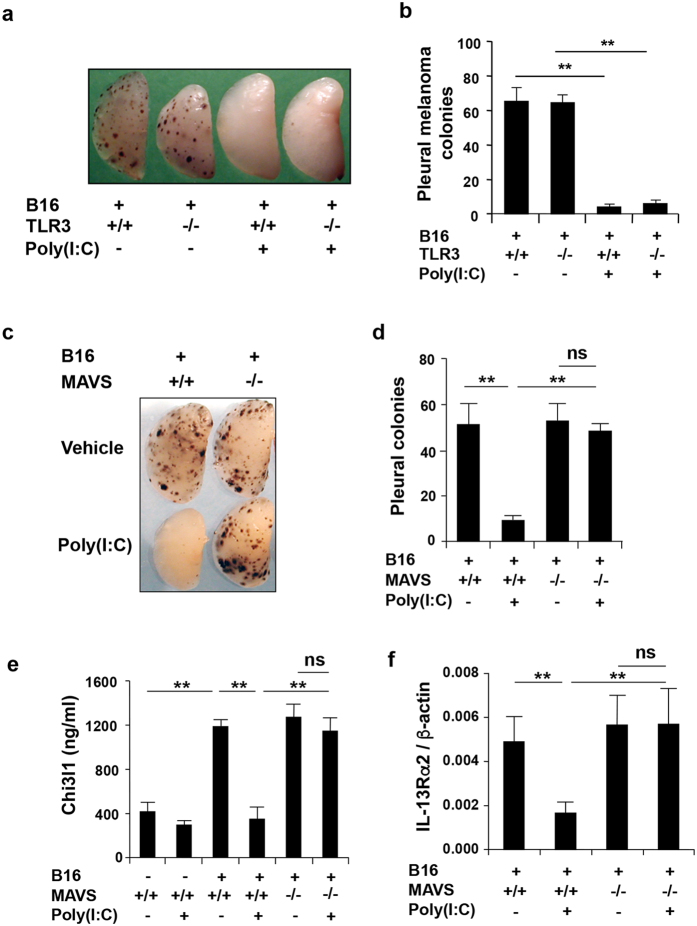
Poly(I:C) inhibits melanoma lung metastasis, Chi3l1 and IL-13Rα2 via a MAVS-dependent and TLR3-independent mechanism(s). (**a,b**) WT, TLR3 null (TLR3^−/−^) or MAVS null (MAVS^−/−^) mice were given B16 melanoma cells (+) or vehicle (−) and treated with Poly(I:C) (+) or control vehicle (−). Lung metastasis was evaluated 2 weeks later. Representative lungs and quantification of pleural melanoma counts in WT (+/+) and TLR3 null mice (−/−). (**c,d**) Representative lungs and pleural melanoma counts in WT (+/+) and MAVS null mice (−/−). (**e,f** ) Quantification of BAL Chi3l1 and whole lung IL-3Rα2 mRNA in WT mice (+/+) and MAVS null (−/−) mice challenged with B16 cells (+) or vehicle control (−). Panels **a** and **c** are representative of a minimum of 5 mice in each group. The values in panels **b**,**d**–**f** represent the mean ± SEM of evaluations with a minimum of 4 mice. **P < 0.01. ns, not significant.

To determine if Poly(I:C) uses innate immunity to inhibit melanoma metastasis, we evaluated the roles of the Toll-like receptor 3 (TLR3) and RIG-helicase (RLH) innate immune pathways. In these experiments, we compared the effects of Poly(I:C) in wild type, TLR3 null and mitochondrial antiviral signaling molecule (MAVS) null mice. MAVS was chosen because it is the central integrator of the RLH pathway that links the viral nucleic acid sensing RNA helicases like retinoic acid inducible gene-I (RIG-I) and melanoma differentiation antigen 5 (Mda5) to anti-viral effector responses^28^,^29^. As noted above, Poly(I:C) was an effective inhibitor of melanoma lung metastasis. Interestingly, Poly(I:C) had a similar ability to inhibit these metastatic responses in TLR3 sufficient and deficient mice (Fig. 2a,b). In contrast, the melanoma inhibitory effects of Poly(I:C) were abrogated in the absence of MAVS (Fig. 2c,d). In accord with the data noted above, the ability of Poly(I:C) to inhibit the accumulation of BAL Chi3l1 and the expression of its receptor IL-13Rα2 were also attenuated in the absence of MAVS but were not altered in the absence of TLR3 (Fig. 2e,f and data not shown). Thus, Poly(I:C) inhibits melanoma metastasis, Chi3l1/BRP-39 accumulation and IL-13Rα2 expression via the RLH innate immune pathway and TLR3 does not play a major role in these responses.

### Metastatic cancer suppresses RLH responses by augmenting the expression of NLRX1 and Poly(I:C) reverses this suppression via a MAVS-dependent mechanism

**Figure 3:**
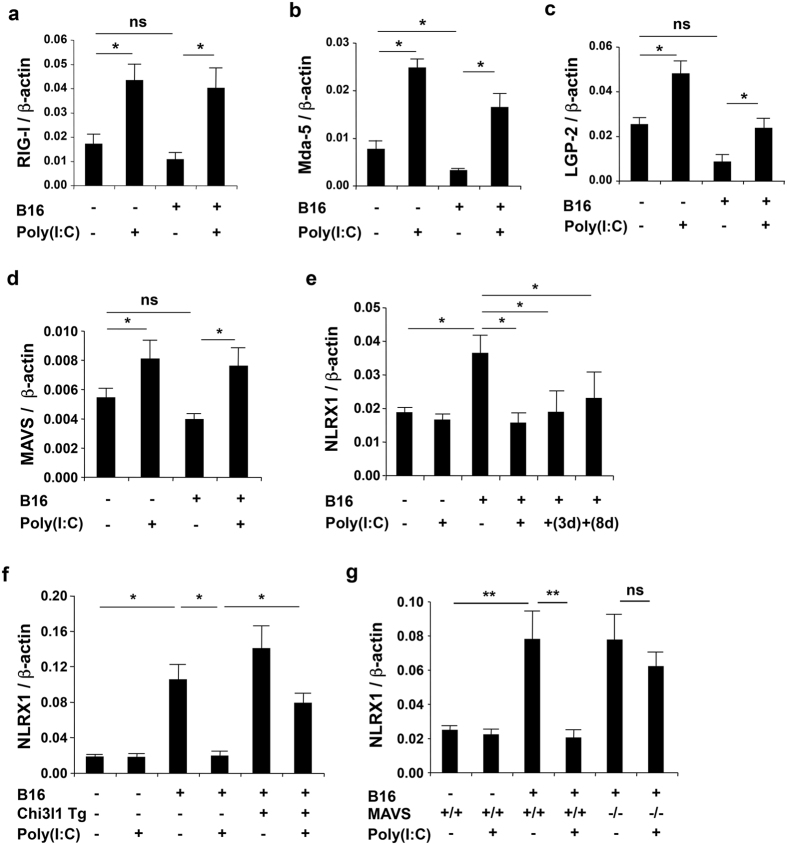
Poly(I:C) reverses the suppression of RLH moieties and augmented expression of NLRX1 caused by pulmonary melanoma metastasis. (**a–e**) WT(+/+) and MAVS null (-/-) mice) mice were given B16 melanoma cells (+) or vehicle (−) and treated with Poly(I:C) or vehicle control. Lung gene expression was evaluated 2 weeks later. The levels of mRNA encoding RIG-I, Mda5, LGP-2, MAVS, and NLRX1 were evaluated by qRT-PCR. In panel **e**, Poly(I:C) was administered one day before (+) or 3 days (3d) or 8 days (8d) after B16 cell administration as noted. (**f,g**) WT (+/+ or non-transgenic (−)), Chi3l1/YKL-40 Tg (+) or MAVS null mutant (−/−) mice were given melanoma cells and treated with Poly(I:C) or vehicle control the levels of NLRX1 mRNA were evaluated by qRT-PCR. The values in the panels represent the mean ± SEM of evaluations in a minimum of 4 mice. *P < 0.05. **P < 0.01. ns, not significant.

The studies noted above demonstrate that RLH pathway activation via MAVS plays an important role in the control of tumor spread. Because virulent tumors have been repeatedly shown to coopt antitumor responses^30^ we hypothesized that the spread of melanoma to the lung could be associated with tumor-induced suppression of RLH activation. We also hypothesized that the antitumor effects of Poly(I:C) are mediated, at least in part, by its ability to reverse these suppressive effects. To test these hypotheses we characterized the expression of MAVS-activating helicases and the RLH/MAVS inhibitor NLRX1 (nucleotide-binding, leucine-rich repeats (NLR) molecule X1 (NLRX1)^31^,^32^,^33^) in unchallenged and tumor challenged mice and the effects of Poly(I:C) on these moieties. These studies demonstrated that the administration of B16 melanoma cells causes modest decreases in the expression of the RIG-I, Mda-5 and LGP-2 helicases and MAVS (Fig. 3a–d). In contrast, tumor challenge caused a significant increase in the expression of NLRX1 (Fig. 3e). Interestingly, Poly(I:C) caused a significant increase in the expression of the all three of the helicases and MAVS (Fig. 3a–d). Importantly, Poly(I:C) also markedly decreased the expression of NLRX1 when administered before or for up to 8 days after the B16 cells (Fig. 3e). These effects of Poly(I:C) were mediated, at least in part by Chi3l1 because the decrease in NLRX1 and increase in RIG-I, Mda5 and LGP2 were significantly decreased in mice in which Chi3l1 was expressed using the CC10 promoter (Fig. 3f and Fig. S1a–c). In all cases, Poly(I:C) augmentation of helicase gene expression and suppression of NLRX1 was mediated via a MAVS-dependent mechanism (Fig. 3g and data not shown). When viewed in combination, these studies demonstrate that the spread of malignant melanoma is associated with decreased expression of essential components of the RLH pathway and heightened expression of the RLH/MAVS inhibitor NLRX1. They also demonstrate that the tumor suppression that is caused by Poly(I:C) is associated with a reversal of these RLH-based metastasis permissive regulatory effects.

### Poly(I:C) induces Type I IFNs which play an essential role in the inhibition of Chi3l1 and melanoma metastasis

**Figure 4:**
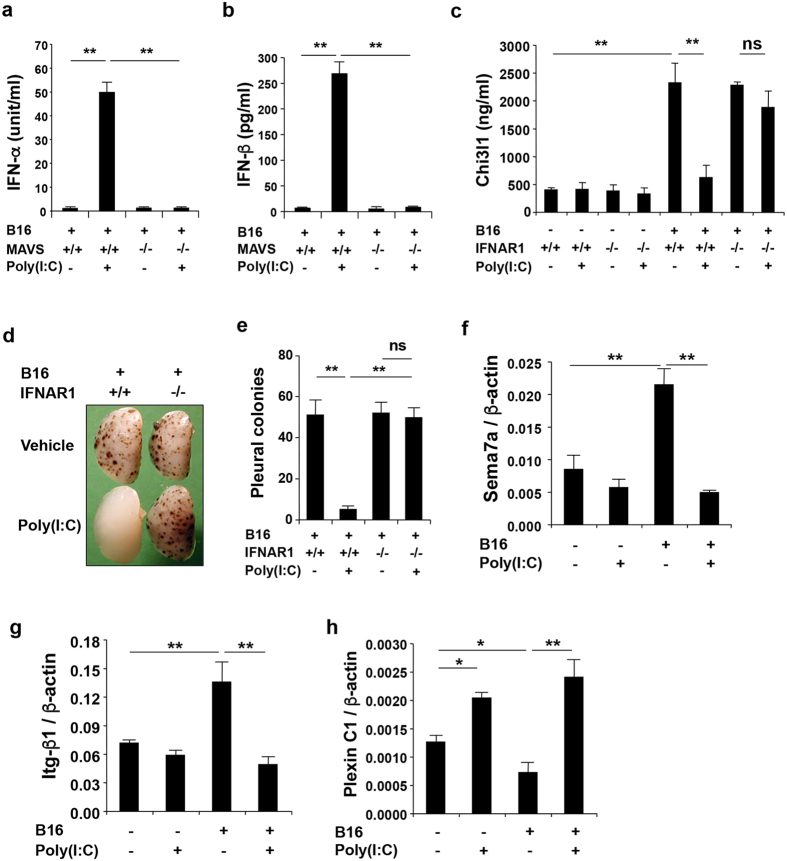
Poly(I:C) stimulates Type I IFNs that inhibit Chi3l1 expression and pulmonary melanoma metastasis and regulates the expression of upstream genes of Chi3l1. WT, MAVS null (MAVS^−/−^) or IFN-α/β receptor 1 null mice (IFNAR1^−/−^) mice were given B16 melanoma cells or vehicle and treated with Poly(I:C) or vehicle. (**a,b**) The levels of IFN-α and IFN-β in BAL fluids from lungs from these mice were evaluated by ELISA. (**c**) BAL Chi3l1 protein was quantitated by ELISA in BAL fluids from lungs from WT (+/+) and IFNAR1 null mice (−/−) that had been given vehicle or melanoma cells and treated with Poly(I:C) or vehicle control. (**d,e**) Representative lungs and pleural melanoma counts in WT (+/+) and IFNAR1 null mice (−/−) that were given melanoma cells and treated with Poly(I:C) or vehicle control. (**f–h**) The levels of mRNA encoding Sema7a, β1 integrin (Itg-β1) and plexin C1 were evaluated by qRT-PCR in the WT mice 2 weeks after B16 melanoma cells or Poly(I:C) treatment. The values in the panels **a–c** and **e–h** represent the mean ± SEM of evaluations with a minimum of 4 mice. Panel **d** is representative of a minimum of 5 similar evaluations. **P < 0.01. ns, not significant.

Because RLH pathway activation has been linked to Type I IFNs, we compared the production of IFN-α and IFN-β in the lungs from mice treated with vehicle or Poly(I:C) and the metastasis-inhibitory effects of RLH activation in WT and Type I IFN receptor (IFNAR1) null mice. Poly(I:C)-induced RLH activation was a potent stimulator of both Type I IFNs in these animals (Fig. 4a,b). These inductive events were MAVS-dependent because they were significantly ameliorated in mice with null MAVS loci (Fig. 4a,b). In addition, Poly(I:C) inhibition of tumor-induced Chi3l1 was also dependent on IFNAR1 (Fig. 4c) and the ability of Poly(I:C)-induced RLH activation to inhibit melanoma metastasis was significantly decreased in mice with null mutations of IFNAR1 (Fig. 4d,e). Thus, Type I IFNs are induced during and play a critical role in the pathogenesis of the RLH-induced inhibition of Chi3l1 and melanoma metastasis.

### RLH activation regulates Sema7a, β1 integrin and Plexin C1

Previous studies from our laboratory demonstrated that melanoma cells stimulate Chi3l1 via a semaphorin 7a (Sema7a)-dependent mechanism with Sema7a stimulating Chi3l1 when it interacts with its β1 integrin receptor and inhibiting Chi3l1 when it interacts with its Plexin C1 receptor^24^. Studies were thus undertaken to determine if RLH activation regulates melanoma metastasis by altering these interactions. Tumor cell challenge increased the expression and accumulation of Sema7a, whereas Poly(I:C)-induced RLH activation inhibited this induction via a MAVS-dependent mechanism (Fig. 4f and data not shown). In addition, tumor cell challenge also stimulated the expression of the β1 integrin receptor while suppressing the Plexin C1 receptor (Fig. 4g,h). Interestingly, Poly(I:C)-induced RLH activation abrogated the induction of β1 integrin and increased the expression of Plexin C1 via a MAVS-dependent mechanism(s) (Fig. 4g,h). These effects were also mediated, at least in part, by Chi3l1 because the ability of Poly(I:C) to inhibit Sema7a and β1 integrin and stimulate Plexin C1 in tumor challenged lungs was significantly ameliorated when Chi3l1 was expressed under the influence of the CC10 promoter (Fig. S1d–f). When viewed in combination, these studies demonstrate that RLH inhibition of Chi3l1 is associated with decreased expression of Sema7a and its Chi3l1-stimulating β1 integrin receptor and the augmented expression of its Chi3l1-inhibiting Plexin C1 receptor.

### RLH activation enhances NK cell accumulation and activation in melanoma challenged lungs

**Figure 5:**
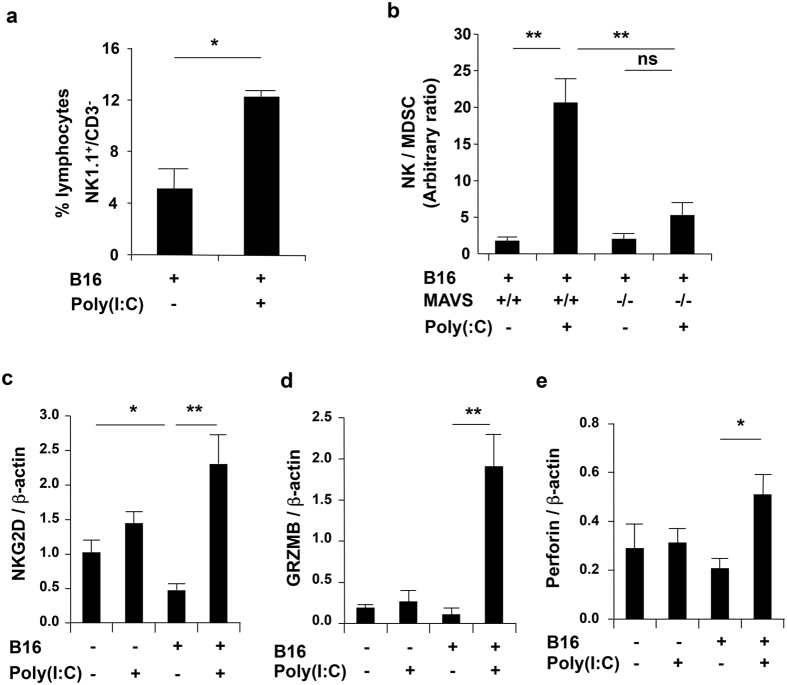
RLH activation enhances NK cell accumulation and activation in melanoma challenged lungs. (**a,b**) WT (+/+) or MAVS null mice (−/−) were given B16 melanoma cells or their vehicle and treated with Poly(I:C) or vehicle control. NK cells were evaluated 2 weeks later. Proportion of NK1.1^+^/CD3^−^ NK lymphocytes and ratio to myeloid derived suppressor cells (MDSC) in lung lysates were evaluated by FACS analysis. (**c**–**e**) mRNA expression of NK cell activating receptor natural killer group 2D (NKG2D), and cytolytic enzymes granzyme B (GRZNB) and perforin in the lungs were evaluated by qRT-PCR. The values in the panels represent the mean ± SEM of evaluations in a minimum of 4 mice. *P < 0.05. **P < 0.01. ns, not significant.

Natural killer (NK) cells have been shown to regulate melanoma growth and metastasis in a number of systems^34^. Thus, studies were undertaken to determine if RLH-mediated tumor suppression was mediated, at least in part, by its ability to alter NK cell accumulation and or function. These studies demonstrated that treatment of tumor challenged mice with Poly(I:C) increased NK cell accumulation (Fig. 5a). The RLH activation was NK cell-specific, at least in part, since no significant changes were noted in the number of CD8 T cells, CD4 T cells and CD19^+^ B cells with and without Poly(I:C) stimulation (Fig. S2). This was seen when expressed as a percentage of lung lymphocytes or as a ratio of NK cells to myeloid-derived suppressor cells (MDSC) (Fig. 5a,b) and was mediated via a MAVS-dependent mechanism (Fig. 5b). B16 cell challenge also caused a significant decrease in the expression of the NK cell activating receptor NKG2D and this inhibition was abrogated by treatment with Poly(I:C) (Fig. 5c). In accord with this finding, RLH activation with Poly(I:C) augmented granzyme B and perforin gene expression in lungs from tumor challenged mice (Fig. 5d,e) and these effects were partially ameliorated when the levels of Chi3l1/YKL-40 were augmented using transgenic methodology (Fig. S1g–i). The NK cell activation was further confirmed by FACS analysis that showed a significant increase in the number of cells expressing NKG2D and peforin or granzyme B with RLH activation by Poly(I:C) stimulation (Fig. S3). These studies demonstrate that RLH activation augments NK cell accumulation and activation in lungs from melanoma-challenged mice via a MAVS-dependent and Chi3l1-regulated pathway.

### Poly(I:C) activates the chemerin pathway via a MAVS-dependent mechanism

**Figure 6:**
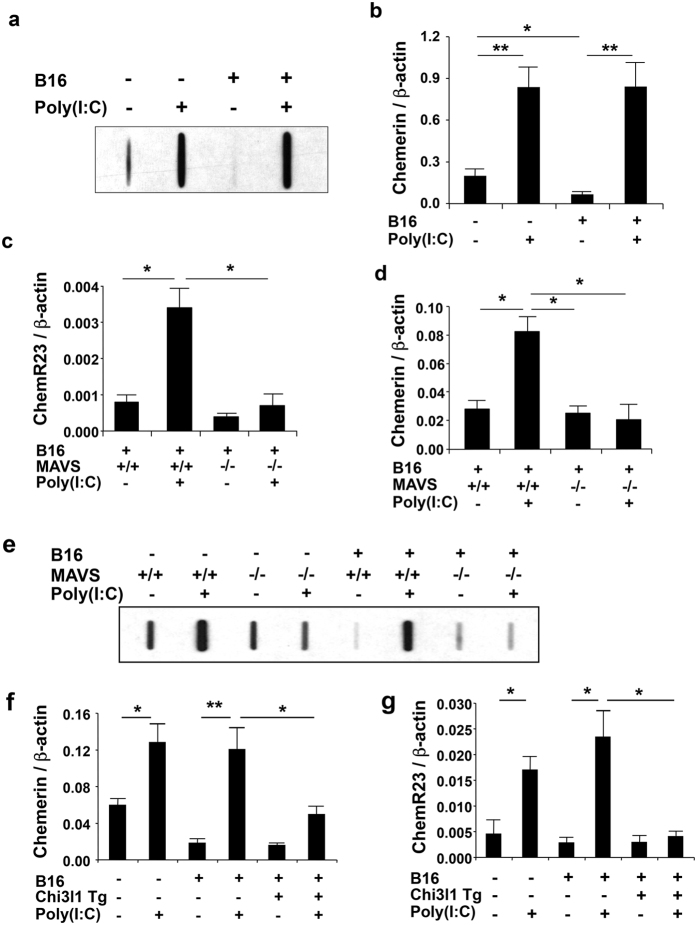
Poly(I:C) activates the chemerin pathway via a MAVS-dependent mechanism. WT (+/+), MAVS null (−/−) or Chi3l1/YKL-40 Tg mice were given B16 melanoma cells or vehicle and treated with Poly(I:C) or its vehicle control. (**a–b**) The expression of chemerin and chemerin receptor (ChemR23) were evaluated 2 weeks later. The levels of chemerin protein and mRNA were evaluated by slot blot assay and qRT-PCR, respectively. (**c,d**) The levels of chemerin receptor and chemerin mRNA in WT and MAVS null mutant mice were evaluated by qRT-PCR. (**e**) The levels of chemerin were evaluated by slot blot assay. (**f,g**) The levels of chemerin and ChemR23 mRNA in WT and Chi3l1 Tg mice detected by qRT-PCR. Panels **a**,**e** are representative of a minimum of 5 mice in each group. The values in the panels **b–d**,**f,g** represent the mean ± SEM of evaluations with a minimum of 4 mice. *P < 0.05. **P < 0.01.

To understand the mechanisms that underlie the augmented NK cell accumulation described above, we evaluated the expression of the endogenous NK cell chemoattractant chemerin in our experimental system. These studies demonstrate that the levels of chemerin accumulation were decreased in bronchoalveolar lavage (BAL) from tumor challenged WT mice (Fig. 6a). They also demonstrate that Poly(I:C)-induced RLH activation augments the levels of chemerin in lungs from unchallenged mice (Fig. 6a,b) and abrogates the inhibition of chemerin and increases the levels of chemerin gene expression in lungs from tumor challenged mice (Fig. 6a,b). In accord with these findings the levels of chemerin receptor 23 (ChemR23, also called CMKLR2) were augmented in tumor challenged mice that were treated with Poly(I:C) (Fig. 6c). In all cases these inductive events were markedly decreased in tissues from mice with null mutations of MAVS (Fig. 6c–e). They were also at least partially Chi3l1-dependent because the ability of Poly(I:C)–induced RLH activation to augment chemerin and chemerin receptor gene expression were significantly decreased when the levels of Chi3l1 were maintained via the transgenic overexpression of Chi3l1/YKL-40 (Fig. 6f,g). These studies demonstrate that chemerin is inhibited in tumor challenged lungs. They also demonstrate that this inhibition is abrogated and that the expression of chemerin and chemerin receptor 23 are increased by RLH activation via a mechanism(s) that involves Chi3l1.

### RLH activation regulates LIM Kinase2, cofilin, PTEN, and BRAF

**Figure 7:**
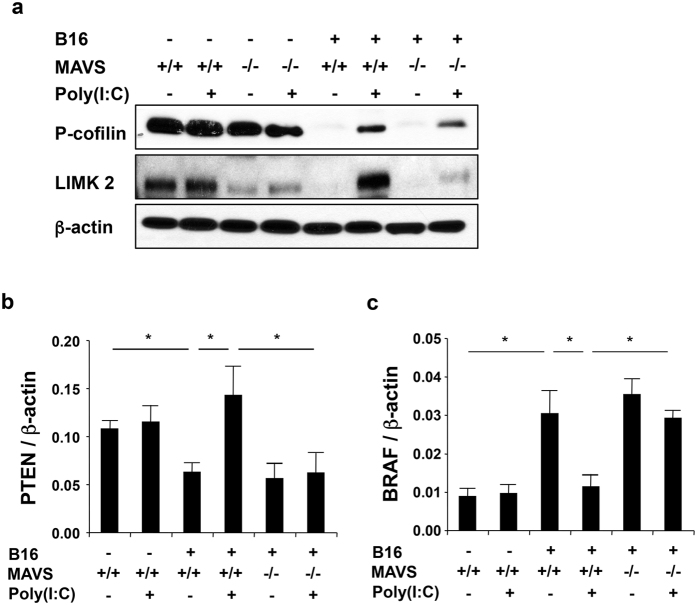
RLH activation regulates LIM kinase2 (LIMK2), cofilin, PTEN, and BRAF. WT (**a**) (+/+) or MAVS null mice (−/−) were given B16 melanoma cells or vehicle and treated with Poly(I:C) or vehicle. Genes associated with the development and progression of malignant melanoma were evaluated 2 weeks later. Western blot evaluation of the activation status of pulmonary cofilin (p-cofilin) and the expression of LIMK2. (**b,c**) The levels of pulmonary PTEN and BRAF mRNA were evaluated by qRT-PCR. Panel **a** is representative of a minimum of 3 independent experiment. The values in panels **b,c** represent the mean ± SEM of evaluations with a minimum of 4 mice. *P < 0.05.

Previous studies demonstrated that melanoma metastasis and invasion are associated with decreased expression of LIM kinase (LIMK) and reduced levels of phosphorylated cofilin (p-cofilin)^35^. Recent studies have also demonstrated that B-Raf proto-oncogene (BRAF) cooperates with phosphatase and tensin homolog (PTEN) loss to induce malignant melanoma^36^. Thus, studies were undertaken to define the regulation of LIMK and p-cofilin, PTEN and BRAF in normal lungs and lungs with metastasis from mice that were treated with Poly(I:C) or vehicle control. As shown in (Fig. 7a), LIMK2 and p-cofilin were readily apparent in lungs from WT mice without metastatic disease and the detection of both was decreased by melanoma metastasis. Interestingly, Poly(I:C)-induced RLH activation partially abrogated these tumor-induced inhibitory effects (Fig. 7a). The expression of the PTEN tumor suppressor was also decreased by melanoma metastasis and this inhibition was abrogated by treatment with Poly(I:C) (Fig. 7b). In contrast, the expression of the BRAF proto-oncogene (the gene that is most commonly mutated in melanoma) was augmented by melanoma metastasis and this stimulation was decreased by Poly(I:C) (Fig. 7c). Importantly, RLH activation suppressed all of these responses and increased LIMK2, p-cofilin and the PTEN tumor suppressor gene while decreasing the expression of BRAF (Fig. 7a–c). In all of these cases, these effects were reduced in tissues from MAVS null mice (Fig. 7a–c). These findings demonstrate that melanoma metastasis is associated with decreased expression of LIMK2 and PTEN, decreased accumulation of p-cofilin and enhanced expression of BRAF and that each of these regulatory events is rescued by RLH activation.

## Discussion

Chi3l1 is produced by and present in elevated quantities in the circulation of patients with a variety of tumors where it is a poor prognostic sign^16^,^37^,^38^,^39^,^40^. In addition, Chi3l1 induction is an essential event in the generation of a metastasis permissive microenvironment^24^. As a result studies were undertaken to define the mechanisms that Chi3l1 uses to foster tumor progression and metastasis. In addition, pathways that regulate tumor induction of Chi3l1 were defined and the effects of these pathways on tumor metastasis were evaluated. Recent studies have suggested that Chi3l1 promotes cancers by altering cell proliferation, macrophage recruitment, angiogenesis and local tumor invasiveness^41^,^42^,^43^,^44^. The present studies add to these findings by demonstrating that Chi3l1 inhibits NK cell accumulation and activation while inhibiting the accumulation of phosphorylated cofilin, LIMK2 and PTEN while stimulating BRAF. Importantly, these studies also demonstrate that RLH activation inhibits Chi3l1 and pulmonary metastasis and that this inhibition is mediated by the ability of RLH activation to inhibit Chi3l1, its receptor IL-13Rα2^11^ and its down stream metastasis permitting cellular, and immune effects via a pathway that acts through Sema7a and its receptors. When viewed in combination these findings allow for the intriguing schema in which RLH activation abrogates pulmonary metastasis by inhibiting Chi3l1 via Sema7a and its receptors which augments NK cell accumulation and activation and regulates p-cofilin, chemerin and its receptor, LIMK2, PTEN and BRAF.

The RLH pathway is believed to play a major role in antiviral responses based on its ability to detect double-stranded (ds) RNA (which is produced during the replication of many viruses) and 5′-triphosphorylated single-stranded RNA (which are produced by many single stranded RNA virus including influenza). The antiviral immune responses are triggered by cytoplasmic RNA sensors including RIG-I and Mda5 which are linked via MAVS to downstream signaling molecules and the induction of Type I IFNs^28^,^29^. Our studies highlight a novel interaction between the evolutionarily conserved chitinase-like proteins and this fundamental and evolutionarily ancient antiviral defense pathway. Specifically, they demonstrate that Poly(I:C)-induced RLH activation inhibits melanoma and other metastasis via a MAVS-dependent mechanism and that the melanoma inhibitory effects of Poly(I:C) are associated with the induction of Type I IFNs and dependent on IFNAR1. These studies are in accord with recent reports highlighting the therapeutic efficacy of Type I IFNs in metastatic melanoma^45^ and recent reports describing the importance of Type I IFNs in cancer immunoediting, a process whereby the immune system suppresses neoplastic growth and shapes tumor immunogenicity by regulating host cell function^46^. Our demonstration that Type I IFNs mediate their anti-melanoma effects, at least in part, by inhibiting the Sema7a-Chi3l1 pathway raises the interesting possibility that the Sema7a-Chi3l1 axis plays an important role in the immunoediting response.

It has recently been appreciated that the RLH innate immune system is finely regulated to allow for appropriate antiviral activation without inappropriate activation and subsequent tissue injury^47^. In the absence of viral infection the system is tonically inhibited by a number of moieties, most notably NLRX1^31^,^32^. After viral infection these inhibitory effects are released allowing an appropriate antiviral response to be initiated^32^. In contrast, when the RLH innate immune response is disinhibited in an inappropriate manner tissue injury is engendered. This can be readily appreciated in studies that demonstrate that exposure to cigarette smoke inhibits NLRX1 expression allowing exaggerated RLH activation and the generation of pulmonary injury and emphysema^47^. The present studies add to our understanding of the importance of tight control of RLH activation by highlighting a novel relationship between pulmonary metastasis and the RLH pathway in cancer. These studies demonstrate for the first time that pulmonary metastasis is associated with RLH pathway suppression that is caused by the exaggerated expression of NLRX1 and decreases in the expression of the helicases that approached and, in the case of Mda5 reached, statistical significance. One can easily see how these alterations could limit the ability of the RLH system to control metastatic spread. Importantly, our studies also demonstrated that Poly(I:C)-induced RLH activation is able to overcome these RLH inhibiting responses by suppressing NLRX1 and augmenting helicase expression thereby allowing the antitumor effects of RLH innate immunity to be appreciated. In keeping with the well known ability of tumor cells to control host antitumor responses^30^, these studies suggest that the ability of tumors to blunt RLH innate immune responses contributes in important ways to the generation of a metastasis permissive host microenvironment. They also suggest that interventions that augment RLH activation or inhibit its inhibitors like NLRX1 may be therapeutically useful in the control of tumor progression. Lastly, they allow for interesting speculations as regards the origins of cancer and the role(s) of viruses in malignant responses.

Chemerin is a chemoattractant protein for NK cells that has been implicated in melanoma homeostasis. It is down-regulated in melanoma cells. In addition, it can elicit antitumor responses via an NK cell dependent mechanism(s) and high levels of expression correlate with improved disease outcomes^34^. Our studies add to our understanding of this system by highlighting a previously unappreciated relationship between chemerin and RLH innate immunity. Specifically they demonstrate that the inhibition of melanoma metastasis that is caused by Poly(I:C)-induced RLH activation is associated with impressive increase in chemerin and NK cell, but not CD8^+^ or CD4^+^ T cell or CD19^+^ B cells, accumulation and that both are mediated by a MAVS- and Chi3l1-dependent mechanism(s). These studies suggest that Chi3l1 inhibition of the chemerin system and NK cell function and activation are important events in the ability of Chi3l1 to foster metastasis and that RLH inhibits pulmonary metastasis, at least in part, by abrogating these NK cell effects.

Previous studies from our laboratory demonstrated that melanoma and other metastasis stimulate Chi3l1 via a novel Sema7a containing pathway in which Sema7a stimulates and inhibits Chi3l1 when it interacts with its β1 integrin and Plexin C1 receptors respectively^24^. The present studies demonstrate that this regulatory apparatus also plays a role in RLH regulation of Chi3l1. Specifically they demonstrate that the increased levels of Chi3l1, enhanced expression of Sema7a and β1 integrins and decreased expression of Plexin C1 that are seen in the setting of pulmonary metastasis are reversed by RLH activation. They also extend this to cofilin, an actin depolymerizing factor that plays critical roles in cell division, chemotaxis and tumor metastasis^48^ and LIMK2 which phosphorylates and inactivates cofilin. The expression of p-cofilin and LIMK2 are both inhibited by tumor metastasis and restored by RLH activation. These studies highlight the important roles that Sema7a and its receptors play in the regulation of Chi3l1 and pulmonary tumor metastasis and the important roles they play as targets of RLH innate immune activation.

PTEN is a powerful multifaceted tumor suppressor that is functionally involved in many different hallmarks of cancer^49^. The majority of its regulatory effects result from its ability to restrain cancer via regulating PI3 Kinase signaling. Loss of PTEN functionality is a critical event in the development of a wide variety of human cancers. Interestingly, a growing body of data supports the contention that that PTEN gene/protein dosage is quantitatively relevant in these responses with partial loss of PTEN function being sufficient to promote human malignancies^49^. During tumor development and progression genetic mutations and transcriptional, epigenetic and post-transcriptional events and protein–protein interactions have been shown to regulate PTEN activity. In keeping with the importance of PTEN in the pathogenesis of melanoma^36^,^50^, our studies demonstrate that pulmonary tumor metastasis is associated with the suppression of PTEN expression. They also demonstrate that RLH activation abrogates this suppression and restores PTEN expression. This raises the interesting possibility that RLH activation could augment the tumor suppressive effects of PTEN and thereby engender therapeutic benefits in a variety of cancers.

The B-Raf oncoprotein is a serine threonine kinase that regulates MAPK/ERK signaling and, in turn, cell growth, differentiation and apoptosis^51^. Mutations of the BRAF gene are common in cancers including lymphomas, melanoma, thyroid cancers, and pulmonary malignancies^51^. In many of these tumors the mutations cause dysregulated binding of RAS to B-Raf and MEK proteins in the Ras/Raf/MEK/ERK cascade causing spontaneous and exaggerated MEK and ERK signaling^52^. Because enhanced BRAF activity plays an important role in the pathogenesis of many types of malignancies, blockers of this pathway have been developed and approved as cancer therapies^51^,^53^. In keeping with this concept our studies demonstrate that B-Raf expression is augmented in the setting of pulmonary metastasis. Importantly, they also demonstrate that RLH activation abrogates this induction via a Chi3l1-dependent mechanism. This raises the interesting possibility that RLH activators or Chi3l1 inhibitors can have antitumor effects that are mediated by their ability to regulate BRAF.

In conclusion, these studies define the many ways that tumor-induced Chi3l1 contributes to the generation of a metastasis permissive microenvironment and highlight the ability of RLH innate immunity to abrogate these responses. Additional investigation of the effects of Chi3l1 in the tumor microenvironment and the ways that RLH innate immunity can be manipulated to control metastasis is warranted.

## Matrials and Methods

### Genetically modified mice

Chi3l1/BRP-39 null mutant (Chi3l1^−/−^) and Chi3l1/YKL-40 transgenic (Tg) mice were generated and characterized in our laboratory as previously described^7^,^54^. Sema7a^−/−^ mice were provided by Dr. A.L. Kolodkin (Johns Hopkins University, Baltimore, MD). TLR3^−/−^ mice were gifts from Richard A. Flavell (Yale School of Medicine, New Haven, CT)^55^. MAVS^−/−^ and IFNAR1^−/−^ mice were provided from Dr. ZJ Chen (University of Texas Southwestern Medical Center, Dallas, Texas)^29^ and R. Enelow (Dartmouth Medical School, Hanover, NH), respectively. All animals were anesthetized with Ketamine/Xylazine (100 mg/10 mg/kg) before any intervention was performed. All experimental procedures were approved by the Institutional Animal Care and Use Committees (IACUC) at Yale and Brown Universities and performed according to the NRC *Guide for the Care and Use of Laboratory Animals* and the Association for Assessment and Accreditation of Laboratory Animal Care (AAALAC).

### Administration of melanoma and Breast cancer cells

The mouse melanoma cell line (B16-F10) established from C57BL6/J mouse melanoma was purchased from ATCC (Cat#: CRL-6475). After being cultured to confluence in Dulbecco’s Modified Eagles Medium (DMEM), the cells were collected, adjusted to a concentrations of 10^6^ cells/ml and delivered to the mice by tail vein injection (2 × 10^5^ cells/mouse in 200 μl of DMEM)^56^. The EMT6 breast cancer cell line was provided by Dr. S. Rockwell (Therapeutic Radiology, Yale School of Medicine). EMT6 cells (2 × 10^4^ cells/moues in 200 μl of DMEM) were delivered via tail vein injection to BALB/c WT mice, because these cells only survive in BALB/c animals^57^.

### Assessment of melanoma and breast cancer pulmonary metastasis

Melanoma lung metastasis was quantified by counting the number of melanoma colonies (which appear as black dots) on the surface of the lung. For the evaluation of EMT6 metastasis, the lungs were harvested, treated with Bouin’s fixative overnight, then washed with 70% alcohol and the number of colonies on the surface of the lungs was counted under a surgical microscope.

### Administration of Poly(I:C)

Poly(I:C) (GE Healthcare Biosciences) was delivered to WT and genetically modified mice via intranasal (i.n.) administration (1 μg/kg/mouse) as described^26^. Unless otherwise stated, Poly(I:C) was administered 24 hours before the tumor cell challenge.

### Assessments of mRNA and protein in the lung

The levels of pulmonary Chi3l1/BRP-39, Sema7a, β1 integrin, Plexin C1, cytokines, RIG-I, Mda-5, LGP-2, MAVS, NLRX1, Type I IFN, granzyme B, perforin, chemerin, ChemR23, p-cofilin, LIMK2, PTEN and BRAF mRNAs and or proteins were assessed using real-time RT-PCR (RT-PCR), Western blotting, ELISA or slot blotting as previously described by our laboratory^7^,^24^,^58^.

### FACS analysis

FACS analysis of NK cells were evaluated as previously described^34^. MSDCs were enumerated as Lin^−^CD11b^hi^Gr-1^+^ cells as previously reported^34^. Plasmacytoid dendritic cells were quantified based on the coexpression of CD11c, B220, and PDCA-1 and major lymphocyte populations were identified as CD3^+^ CD4^+^ CD8^−^, CD3^+^ CD4^−^, CD8^+^ and CD3^−^ CD19^+^ as previously described^34^.

### Immunoblot analysis

Whole-lung lysates were prepared and the total protein content of each was measured using the DC protein assay reagents (Bio-Rad, Hercules, CA). Equal amounts of sample proteins were fractionated on 4%–15% SDS-PAGE gels under reducing conditions. These were individual gels that were prepared and run at the same time. The sample proteins were transferred to polyvinylidene difluoride membranes and incubated in blocking buffer (5% w/v nonfat dry milk in TBS/0.05% Tween 20) for 1 hour at room temperature. They were then incubated with primary antibodies overnight at 4 °C, washed 3 times in TBS/0.05% Tween 20, and incubated for 2 hours at room temperature with appropriate secondary antibodies. Immunoreactive signal was detected using a chemiluminescent procedure (ECL Western blotting detection system; GE Healthcare Biosciences, Piscataway, NJ) according to the manufacturer’s instructions.

### Statistics

Statistical evaluations were undertaken with SPSS software. As appropriate, groups were compared with 2-tailed Student’s *t* test or with nonparametric Mann-Whitney *U* test. Values are expressed as mean ± SEM. Statistical significance was defined as a level of *P* < 0.05.

## Additional Information

**How to cite this article**: Ma, B. *et al*. RIG-like Helicase Regulation of Chitinase 3-like-1 Axis and Pulmonary Metastasis. *Sci. Rep.* **6**, 26299; doi: 10.1038/srep26299 (2016).

## Supplementary Material

Supplementary Information
