# Supplementary material for: RIG-like Helicase Regulation of Chitinase 3-like 1 Axis and Pulmonary Metastasis
Source: Sci Rep. 2016 May 20;6:26299. doi: 10.1038/srep26299 (PMC4873814; doi:10.1038/srep26299)
Supplement: Supplementary Information [file srep26299-s1.pdf]

Supplemental Information

RIG-like Helicase Regulation of Chitinase 3-like-1 Axis and Pulmonary Metastasis

Bing Ma., Erica L. Herzog, Meagan Moore, Chang-Min Lee, Sung Hun Na, Chun Geun Lee, and Elias, J.A

Supplementary Figures S1-S3

Supplementary Figure legends :

Fig. S1. Effects of transgenic Chi3l1/YKL-40 on Poly(I:C) regulation of pulmonary RLH, Chi3l1-regulating genes and NK cell genes.
WT and Chi3l1/YKL-40 Tg mice were given B16 melanoma cells or control vehicle, treated with Poly(I:C) or vehicle control and evaluated 2 weeks later. The levels of pulmonary mRNA encoding the noted genes were evaluated by RT-PCR. The plotted values represent the mean± SEM of evaluations with a minimum of 4 mice. *$P<0.05$. **$P<0.01$

Fig. S2. RLH regulation of CD4, CD8 T cells and CD19+ B cells in melanoma lung metastasis.
WT and Chi3l1/YKL-40 Tg mice were given B16 melanoma cells, treated with Poly(I:C) or vehicle control and the distribution of CD8 T cells, CD4 T cells and B cells in the lung was evaluated 2 weeks later by FACS analysis. (a and b) % and number of CD8 T cells (CD3+, CD4-, CD8+). (c and d) % and number of CD4 T cells (CD3+, CD4+, CD8-). (e and f) % and number of B cells (CD3-, CD19+). The plotted values represent the mean±SEM of evaluations with a minimum of 4 mice. ns, non significant.

Fig. S3. RLH regulation of NK cell activation. FACS analysis on the cells expressing NKG2D and perforin or granzyme B in the lung of melanoma metastasis with and without Poly(I:C) stimulation. (a) Cells expressing NKG2D and perforin (% and number of $NKG2D^+/Perforin^+$ cells). (b) Cells expressing NKG2D and granzyme B (% and number of $NKG2D^+$/Granzyme B^+ cells). pIC, poly (I:C). The plotted values represent the mean± SEM of evaluations with a minimum of 4 mice. ns, non significant. *$p<0.05$.

Supplementary Figures

Fig. S1

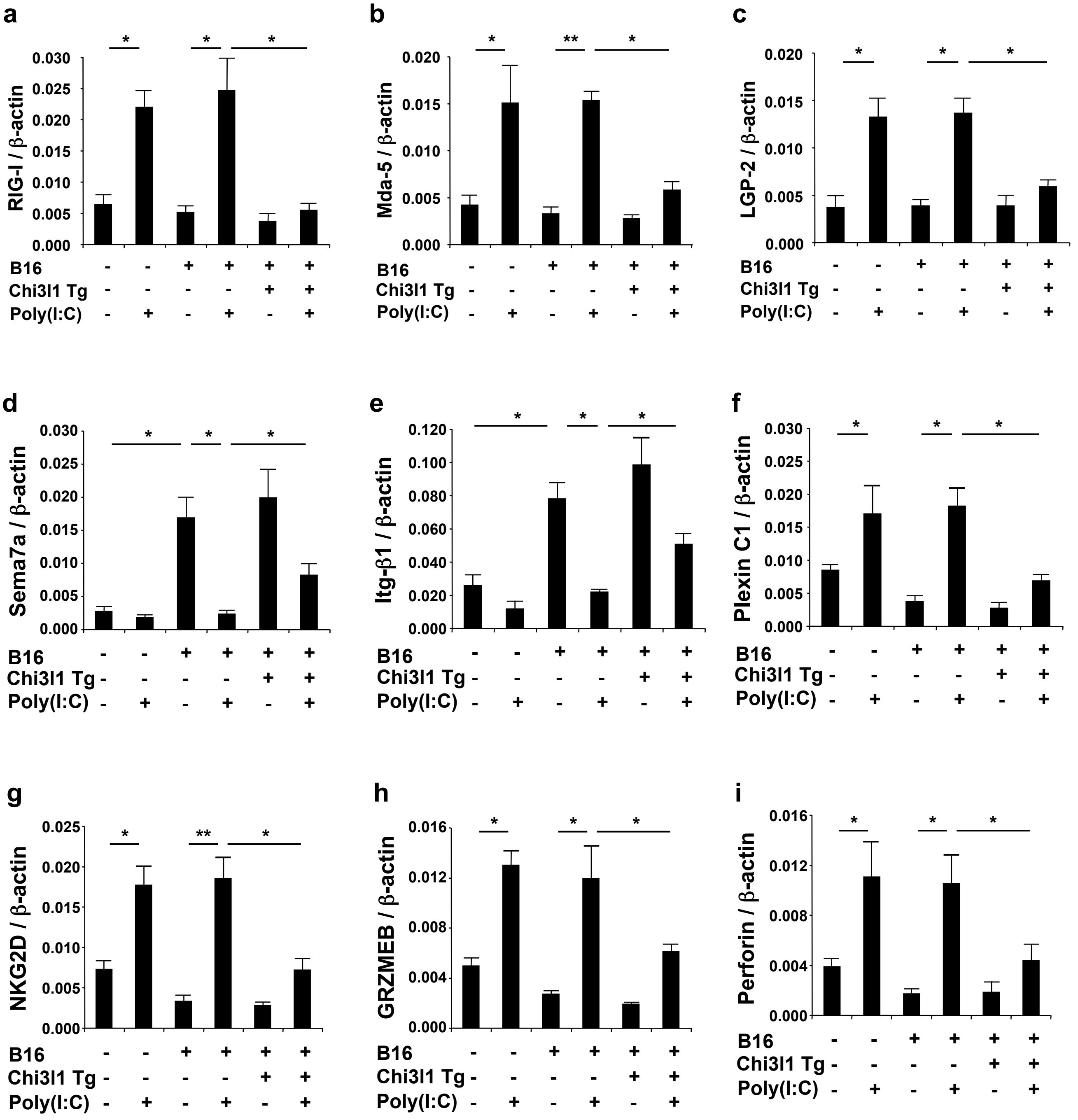

Fig. S2

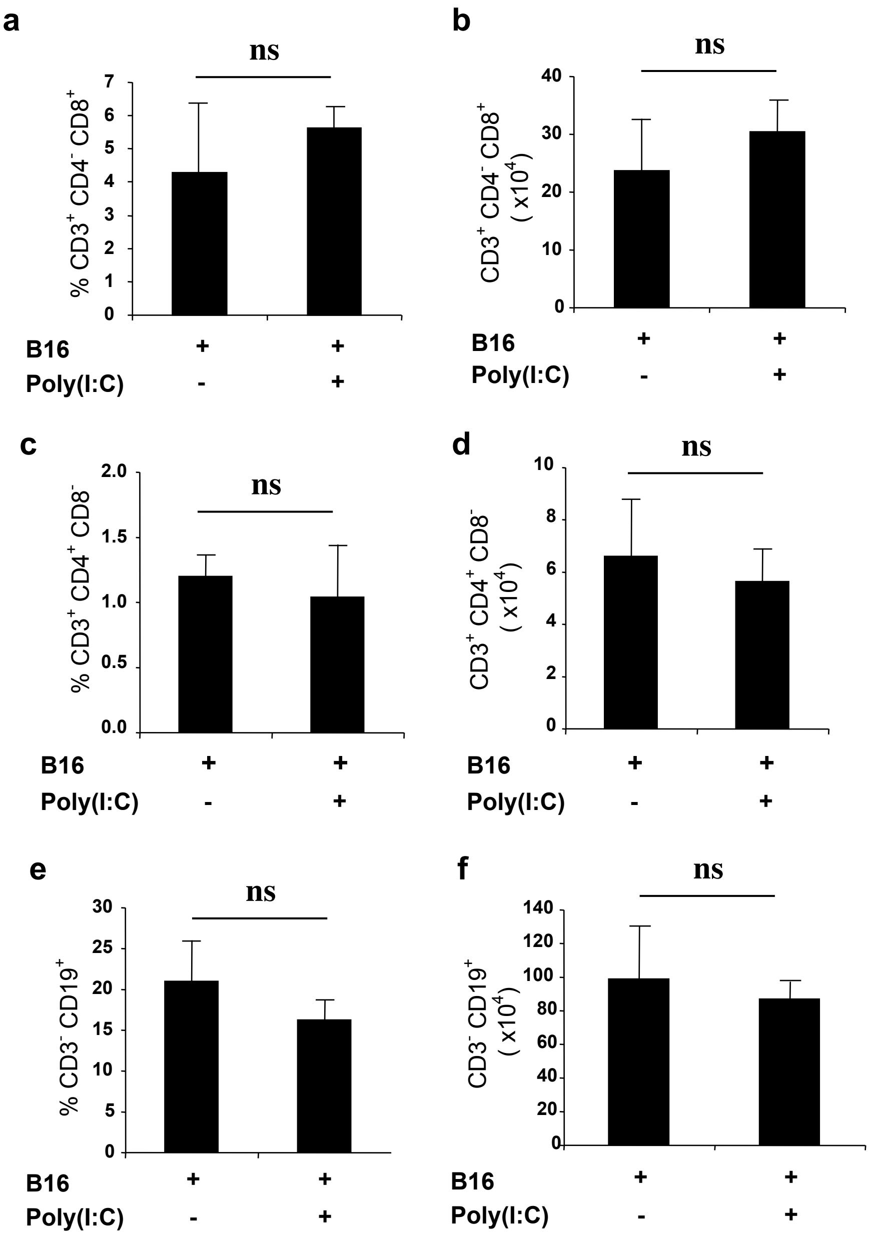
a
ns
% CD3+ CD4- CD8+
0 1 2 3 4 5 6 7
B16 + +
Poly(I:C) - +
b
ns
CD3+ CD4- CD8+ (x10^4)
0 10 20 30 40
B16 + +
Poly(I:C) - +
c
ns
% CD3+ CD4+ CD8-
0.0 0.5 1.0 1.5 2.0
B16 + +
Poly(I:C) - +
d
ns
CD3+ CD4+ CD8- (x10^4)
0 2 4 6 8 10
B16 + +
Poly(I:C) - +
e
ns
% CD3- CD19+
0 5 10 15 20 25 30
B16 + +
Poly(I:C) - +
f
ns
CD3- CD19+ (x10^4)
0 20 40 60 80 100 120 140
B16 + +
Poly(I:C) - +

Fig. S3

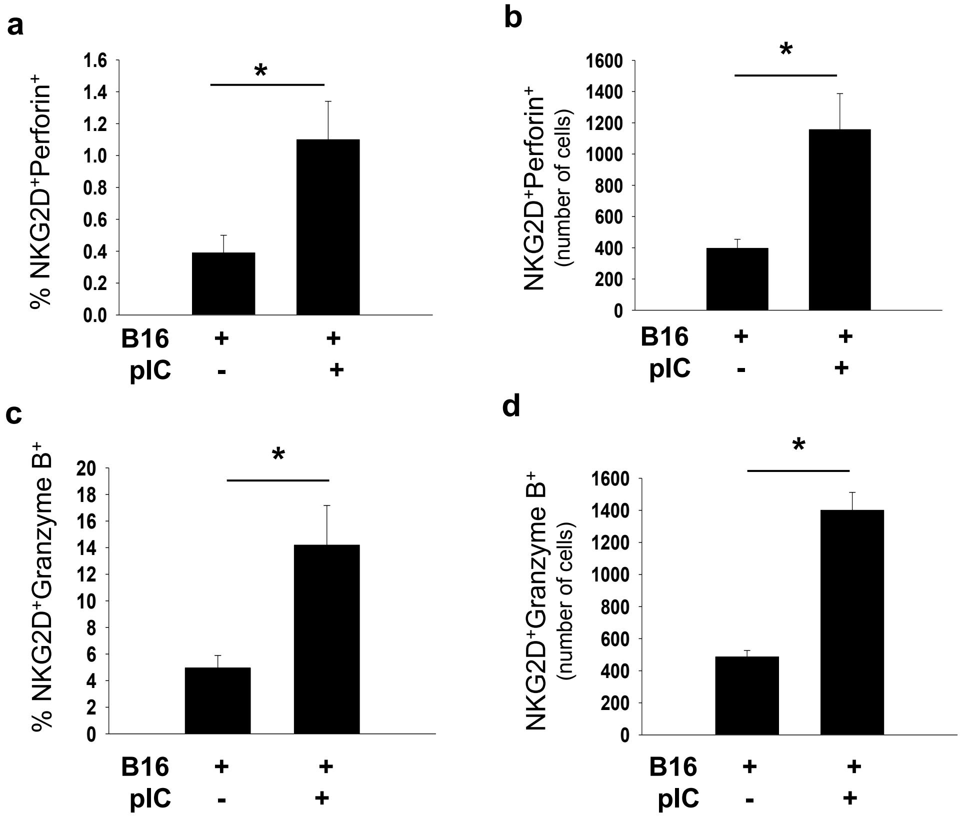

a
% NKG2D+Perforin+
1.6
1.4
1.2
1.0
0.8
0.6
0.4
0.2
0.0
*
B16 + +
pIC - +
b
NKG2D+Perforin+
(number of cells)
1600
1400
1200
1000
800
600
400
200
0
*
B16 + +
pIC - +
c
% NKG2D+Granzyme B+
20
18
16
14
12
10
8
6
4
2
0
*
B16 + +
pIC - +
d
NKG2D+Granzyme B+
(number of cells)
1600
1400
1200
1000
800
600
400
200
0
*
B16 + +
pIC - +
